# Endoscopic Ultrasound-Guided Versus Percutaneous Transhepatic Biliary Drainage in Patients With Malignant Biliary Obstruction: Which Is the Optimal Cost-Saving Strategy After Failed ERCP?

**DOI:** 10.3389/fonc.2022.844083

**Published:** 2022-02-25

**Authors:** Won Jae Yoon, Eric D. Shah, Tae Hoon Lee, Sunguk Jang, Ryan Law, Do Hyun Park

**Affiliations:** ^1^ Department of Internal Medicine, Ewha Womans University College of Medicine, Seoul, South Korea; ^2^ Section of Gastroenterology and Hepatology, Dartmouth-Hitchcock Medical Center, Lebanon, NH, United States; ^3^ Department of Internal Medicine, Soonchunhyang University College of Medicine, Cheonan, South Korea; ^4^ Department of Gastroenterology and Hepatology, Cleveland Clinic, Cleveland, OH, United States; ^5^ Division of Gastroenterology and Hepatology, Mayo Clinic, Rochester, MN, United States; ^6^ Digestive Diseases Research Center, Department of Internal Medicine, University of Ulsan College of Medicine, Asan Medical Center, Seoul, South Korea

**Keywords:** endoscopic ultrasound, percutaneous transhepatic biliary drainage, ERCP, biliary tract obstruction, medical cost

## Abstract

**Background and Aim:**

Although endoscopic ultrasound-guided biliary drainage (EUS-BD) after failed primary ERCP in malignant distal biliary obstruction has similar clinical outcomes compared to percutaneous transhepatic biliary drainage (PTBD), little is known about optimal cost-saving strategy after failed ERCP. We performed a cost analysis of EUS-BD and PTBD after failed ERCP in two countries with different health care systems in the East and West.

**Methods:**

From an unpublished database nested in a randomized controlled trial, we compared the cost between EUS-BD and PTBD in Korea. The total cost was defined as the sum of the total biliary drainage costs plus the cost of hospital stay to manage adverse events. We also performed a cost-minimization analysis using a decision-analytic model of a US Medicare population.

**Results:**

In Korea, the median total costs for the biliary intervention ($1,203.36 for EUS-BD vs. $1,517.83 for PTBD; *P*=.0015) and the median total costs for the entire treatment were significantly higher in PTBD ($4,175.53 for EUS-BD vs. $5,391.87 for PTBD; *P*=.0496) due to higher re-intervention rate in PTBD. In cost-minimization analysis of US Medicare population, EUS-BD would cost $9,497.03 and PTBD $13,878.44 from a Medicare insurance perspective (average cost-savings in choosing EUS-BD of $4,381.41 in the US). In sensitivity analysis, EUS-BD was favored over PTBD regardless of the expected re-intervention rate in EUS-BD and PTBD.

**Conclusions:**

EUS-BD may have an impact on cost-savings due to better clinical outcomes profile compared to PTBD after failed ERCP, even in different medical insurance programs.

## Introduction

The standard management of unresectable malignant distal biliary obstruction (MDBO) is endoscopic retrograde cholangiopancreatography (ERCP) with biliary drainage (BD) (1–3). However, ERCP fails in about 5% to 7% (35,000–49,000) of 700,000 ERCP cases performed annually in the US (4), and percutaneous transhepatic BD (PTBD) has been the standard procedure for the biliary decompression in such cases with MDBO (5). Therefore, failed ERCPs may result in significant costs and healthcare burden. In a recent study (6), 44.7% of failed ERCPs were referred to interventional radiology, which resulted in longer length of hospital stay and higher procedure costs than undergoing repeat ERCP. Salvage interventional procedures with lower costs may result in the reduction of the costs associated with failed ERCPs.

Since the first introduction of endoscopic ultrasound (EUS)-guided choledochoduodenostomy in 2001 (7), EUS-guided BD (EUS-BD) has gained popularity for biliary decompression when ERCP fails. In 2016, we published the results of a multicenter, randomized controlled clinical trial comparing the efficacy of EUS-BD and PTBD after failed primary ERCP in unresectable MDBO. The results showed that EUS-BD and PTBD had similar efficacy and quality of life. Of note, EUS-BD was superior to PTBD in terms of the rate of procedure-related adverse events and unscheduled re-interventions (8). A meta-analysis also demonstrated that EUS-BD after a failed ERCP was associated with a better clinical success rate, lower rate of adverse events and fewer reinterventions (9). As the need for unscheduled re-intervention often stems from the concern of catheter/tube malfunction, or active infection, a wide range of clinical implications exists with unscheduled re-intervention: from empiric use of antibiotics to unplanned hospitalization of the patients. In addition, a patient survey showed that patients preferred EUS-BD if expertise was available, and the adverse rate was lower than that of PTBD (10).

Until now, optimal cost-saving strategy of biliary decompression after failed ERCP in patients MDBO has not been fully evaluated. To explore the impact of EUS-BD in this aspect, we performed cost comparison of EUS-BD and PTBD in the management of failed ERCP in these patients in countries with different health care systems.

## Methods

### Study Design

Due to significant differences between Korea and the US in medical insurance programs (such as fee-for-service vs. capitated payment) and patient management (such as length of stay and timing/frequency of consultation), separate models and analytic methods were performed for each country as appropriate. Each model assumed inputs that were appropriate to the needs of calculating reimbursement in each country.

#### Cost Comparison of the BD Strategies in Korea From a Previous Randomized Controlled Trial

A multicenter, prospective, randomized, controlled, non-inferiority trial comparing the efficacy of EUS-BD and PTBD after failed ERCP was published previously in 2016 (8). The primary endpoint was technical success. The secondary endpoints were functional success, procedure-related adverse events, the rate of unscheduled re-intervention, and quality of life. We had also collected data on the costs of the procedures, which was not presented in the publication (8).

From the available data, we compared the cost between EUS-BD and PTBD. The total cost of the entire treatment was defined as the sum of the reimbursement and non-reimbursement costs for EUS-BD (fee-for-service and device costs including those of an FNA needle, a guidewire, dilation device, and a metal stent) or PTBD (PTBD catheter insertion with or without transpapillary metal stent placement, and the removal of PTBD catheter as fee-for-service and device costs) as the primary BD plus the costs of hospital stays with the management of the adverse events of each treatment approach as an unscheduled biliary re-intervention. The costs of each procedure and daily hospital charges were converted from Korean won to US dollars according to the annual average exchange rate and an annual medical fee schedule of National Health Insurance in Korea (11). The unit costs and their sources are shown in **Table 1**.

**Table 1:** Overview of cost calculations for interventions in US dollars^a^.

KOREA	
Cost category	Costs
**Charge for primary interventions**	
ERCP	413.42
EUS-BD^b^	1028.27
PTBD	762.80
Second PTBD for metal stent insertion	350.82
**Secondary interventions**	
Second ERCP	206.76
PTBD, another site	762.80
Tubography	141.39
PTBD tube change	292.78
**Basic hospital cost per day** ^c^	74.25
**UNITED STATES**	
**Cost category**	**Costs**
EUS-BD Includes physician billing based on CPT 99285 (emergency department visit), CPT 99223 (initial hospital care), CPT 99238 (hospital discharge day), CPT 99223 (initial hospital care for gastroenterology consultation), CPT 99232 (subsequent hospital care for consultation), hospital billing based on DRG 445 (Disorders of the biliary tract, with at least one complication/comorbidity), and procedural billing based on an MRI/MRCP (CPT 74183), ERCP (CPT 43260), and EGD (CPT 42340) with anesthesia (CPT 00732 coded in 15-minute units * 6 standard increments)	8,002.48
Re-intervention with PTBD after failed EUS-BD *Includes additional physician billing based on CPT 99223 (initial interventional radiology consultation), 99233 (subsequent hospital care), CT abdomen (CPT 74178), placement of a percutaneous biliary drainage catheter (CPT 47533), and subsequent outpatient percutaneous conversion of a catheter to a stent (CPT 47538 + APC 5361)*	6,351.83
PTBD *Includes physician billing based on CPT 99285 (emergency department visit), CPT 99223 (initial hospital care), CPT 99238 (hospital discharge day), CPT 99223 (initial hospital care for gastroenterology and interventional radiology consultations), CPT 99232 (subsequent hospital care for gastroenterology consultation), hospital billing based on DRG 445 (Disorders of the biliary tract, with at least one complication/comorbidity), and procedural billing based on an MRI/MRCP (CPT 74183), the initial failed ERCP (CPT 43260), placement of a percutaneous biliary drainage catheter (CPT 47533), and subsequent outpatient percutaneous conversion of a catheter to a stent (CPT 47538 + APC 5361)*	13,369.98
Re-intervention after failed PTBD *Includes additional physician billing based on subsequent hospitalist, gastroenterology and interventional radiology consultation (CPT 99233), CT abdomen (CPT 74178), and subsequent placement of a percutaneous biliary drainage catheter (CPT 47533)*	957.11
**Outcomes category**	**Outcome**
Re-intervention rate after EUS-BD Re-intervention rate after PTBD	23.6% (beta distribution in probabilistic sensitivity analysis on binomial data*; ranged from 0-100% in one-way sensitivity analysis) 53.1% (beta distribution in probabilistic sensitivity analysis on binomial data*; ranged from 0-100% in one-way sensitivity analysis)

ERCP, endoscopic retrograde cholangiopancreatography; EUS-BD, endoscopic ultrasound-guided biliary drainage; PTBD, percutaneous transhepatic biliary drainage; CPT, computerized procedural terminology; APC, ambulatory payment classification. 2021 conversion factors were used for US costs according to the Centers for Medicare and Medicaid Services. Cost estimates were derived from 2021 US Centers for Medicare and Medicaid Services data including the Physician Fee Schedule, Inpatient Prospective Payment System, and Hospital Outpatient Prospective Payment System similar to previous cost analyses (https://www.cms.gov) (12).

*Reference (8).

a Costs converted from Korean won to Us dollars according to medical fee schedule of National Health Insurance of Korea in 2014.

b The cost of EUS-BD includes fee-for-service and device costs including the those of an FNA needle, a guidewire, dilation device, and a metal stent.

c Basic cost for patient room and diet.

^c^Underlined word is to emphasize billing.

#### Cost-Minimization Analysis for the Cost Comparison of the BD Strategies of US Medicare Population

The number of patients in which rate of ERCP failed and number of PTBD/EUS-BD were assumptions of the model that determine cost as the output in US cost analysis. The base case US patient was a 65-year-old Medicare-eligible patient admitted to the hospital for painless jaundice having already undergone one ERCP that failed to achieve BD (see **Table 1** for model inputs). Age 65 is when patients are eligible for US Medicare which is standard to anchor this type of analysis to improve generalizability of findings. We evaluated the potential cost savings for the following BD procedures: (1) proceed to EUS-BD, or (2) proceed to PTBD, followed by stent internalization and the removal of PTBD catheter as an outpatient by interventional radiologists. The rates of re-interventions were extracted from a previous randomized controlled trial (8), and we assumed that rates of technical success in achieving BD were included in rates of re-intervention to avoid double-counting of these outcomes (8). For the purposes of our analysis, we also assumed that no deaths or serious adverse events would be typically expected and that less serious adverse events were already included in rates of re-intervention, consistent with patient outcomes in the same clinical trial used in the cost-analysis performed on a Korean population (8).

### Statistical Methods

#### Cost Comparison in the Korean Study

Data are presented as median (range). Continuous variables were compared using the Mann-Whitney test. Given the sample size, an automated stepwise variable selection method performed on 1,000 bootstrap samples was also used to provide the cost difference between two modalities with 95% confidence interval to approximate the expected values from the general population. A probability level of *P*<.05 was considered statistically significant. All analyses were performed using STATA/SE (version 14.0, StataCorp, College Station, TX, USA).

#### Cost Comparison in the US Medicare Population

To understand differences in costs between each management strategy, a Markov model was developed to evaluate healthcare costs from a Medicare insurance perspective consistent with the CHEERS checklist and Second Panel on Cost-Effectiveness in Health and Medicine (13). A time horizon of 30 days (and no discount rate) was used to model immediate differences in reimbursement and to give greater weight toward immediate technical challenges and postoperative adverse events, recognizing that a longer time horizon would increasingly favor EUS-BD due to the need for tube exchange with PTBD. Cost-minimization analysis was performed using base-case assumptions to evaluate the primary outcome of cost associated with each strategy. Probabilistic sensitivity analysis was performed using a Monte Carlo of 10,000 trials to model uncertainty in overall cost estimates (reported as 95% confidence intervals for base-case outcomes). One-way sensitivity analysis was performed to assess how cost preferences might be affected by varying the expected rate of any necessary re-intervention following either EUS-BD or PTBD. Analyses were performed using TreeAge Pro 2020 (TreeAge Software, Williamstown, MA, USA).

All authors had access to the study data and had reviewed and approved the final manuscript.

## Results

### Cost Comparison of EUS-BD and PTBD After Failed ERCP in Korea

The results of cost-analysis are shown in **Table 2**. The median hospital charges other than BD ($3,018.78 in the EUS-BD group vs. $3,612.65 in the PTBD group; *P*=.1471) and the median costs of unscheduled reintervention ($174.21 in the EUS-BD group vs. $340.62 in the PTBD group; *P*=.2583) were not significantly different between the two groups. The median costs of primary BD were higher in the PTBD group than the EUS-BD group ($1,029.15 in the EUS-BD group vs. $1,177.21 in the PTBD group; *P*=.0001). The median total costs for the biliary intervention were higher in the PTBD group ($1,203.36 in the EUS-BD group vs. $1,517.83 in the PTBD group; *P*=.0015) as well. The median total costs for the entire treatment were also significantly higher in the PTBD group ($4,175.53 in the EUS-BD group vs. $5,391.87 in the PTBD group; *P*=.0496).

**Table 2:** Cost comparison of EUS-BD and PTBD in Korea.

	EUS-BD	PTBD	P-value
Hospital charges other than biliary drainage	3,018.78 (1,036.13-9,107.64)	3,612.65 (1,154.37-13,092.19)	.1471
Cost of primary biliary drainage intervention	1,029.15 (1,029.15-1,313.97)	1,177.21 (763.44-1,876.01)	.0001
Cost of unscheduled re-intervention for biliary drainage	174.21 (0-1,649.80)	340.62 (0-1,792.59)	.2583
Total cost of biliary drainage interventions	1,203.36 (1,029.15-2,963.76)	1,517.83 (1,177.21-2,969.79)	.0015
Total cost	4,175.53 (2,065.28-10,343.67)	5,391.87 (2,505.12-14,269.40)	.0496

EUS-BD, endoscopic ultrasound-guided biliary drainage; PTBD, percutaneous transhepatic biliary drainage.

Values in median (range).

Cost in US dollars.

This cost analysis was based on the database from a randomized trial (a total of 66 patients [34 patients in the EUS-BD group and 32 patients in the PTBD group]) (8). Study protocol including the cost analysis is available at https://www.cghjournal.org/article/S1542-3565(15)01716-4/fulltext#relatedArticles.

The number of procedures in primary BD of the PTBD group was higher than that of the EUS-BD group (all 32 [100%] patients with one session in EUS-BD vs. 15 [48.4%] of 31 patients with two or three sessions in the PTBD group [PTBD insertion, metal stent placement through percutaneous tract in separate session, and the removal of PTBD tube] and the remaining 16 patients leaving PTBD tube in place for continuous external drainage). The re-intervention rate and the mean re-intervention frequency were higher in the PTBD group (re-intervention rate of 25% in the EUS-BD group and 54.8% in the PTBD group, *P*=.015; the mean re-intervention frequency of 0.34 per patient in the EUS-BD group and 0.93 per patient in the PTBD group, *P*=.02) (8).

The bootstrap analysis of the difference in the costs for intervention with EUS-BD and PTBD is shown in the **Table 3**. EUS-BD was associated with the cost-savings of $1488.35 compared to PTBD in terms of total costs for the entire treatment (**Table 3**).

**Table 3:** Bootstrapped bias-corrected 95% confidence intervals for the cost difference between EUS-BD and PTBD (cost of EUS-BD minus that of PTBD).

	Cost difference	95% confidence interval
Hospital charges other than biliary drainage	-1194.24	-2408.66, -36.34
Cost of primary biliary drainage intervention	-122.85	-206.87, -50.23
Cost of unscheduled re-intervention for biliary drainage	-171.26	-371.57, 27.91
Total cost of biliary drainage interventions	-294.10	-497.90, -71.17
Total cost	-1488.35	-2672.03, -249.27

EUS-BD, endoscopic ultrasound-guided biliary drainage; PTBD, percutaneous transhepatic biliary drainage.

Cost in US dollars.

### Cost Comparison of EUS-BD and PTBD After Failed ERCP in the US

**Figure 1:**
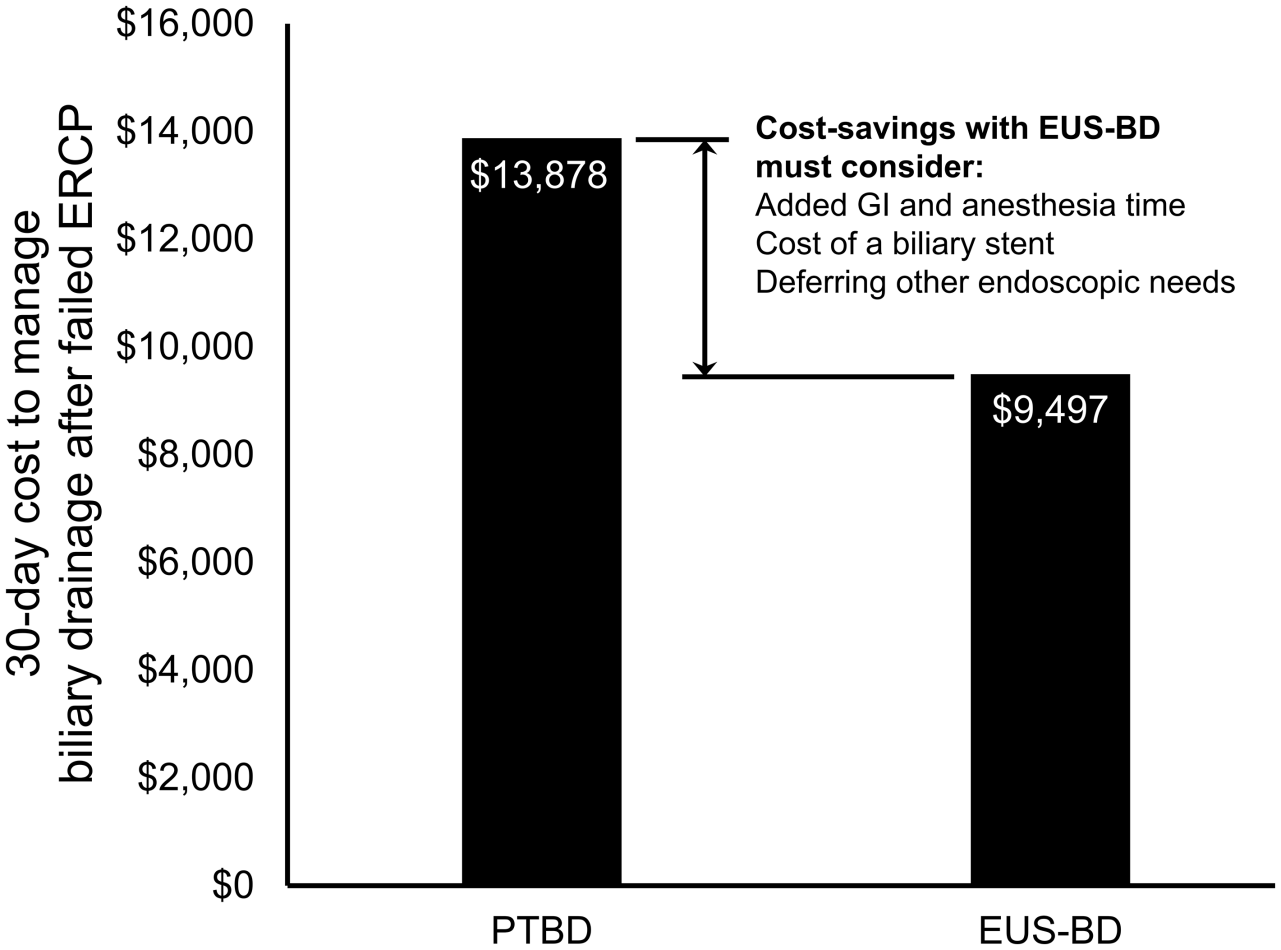
Total US Medicare reimbursement (i.e., costs to Medicare) with EUS-BD compared to PTBD. In base-case analysis, EUS-BD is cost-saving to Medicare compared to PTBD to achieve successful biliary drainage due to a biliary obstruction in patients with a failed ERCP. EUS-BD, endoscopic ultrasound-guided biliary drainage; PTBD, percutaneous transhepatic biliary drainage; ERCP, endoscopic retrograde cholangiopancreatography.

**Figure 2:**
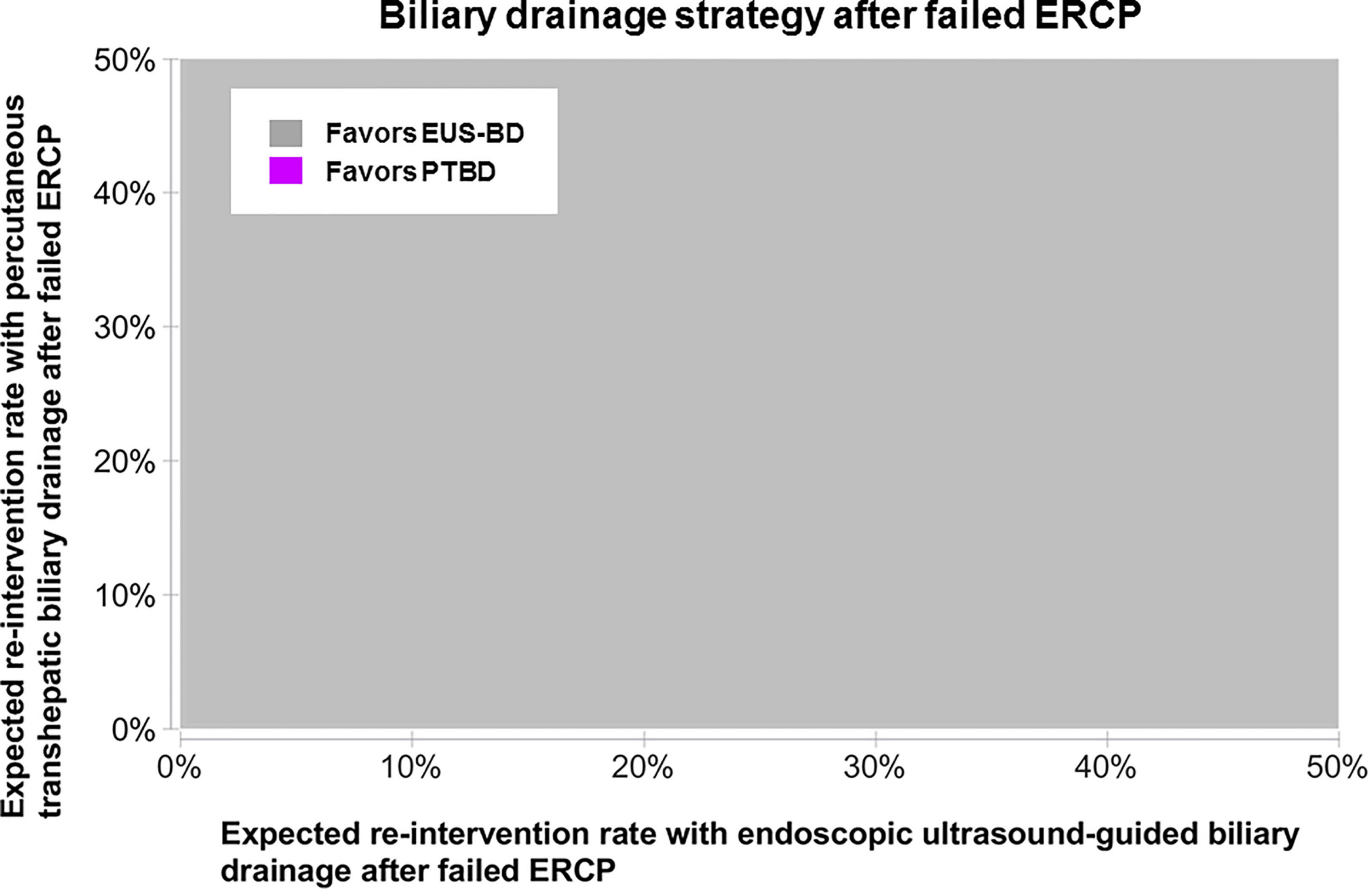
Sensitivity analysis to determine the favored approach on cost-minimization basis depending on the expected re-intervention after EUS-BD or PTBD in a patient who failed ERCP. The concept of this plot is to highlight the preferred strategy at any chosen point along x- and y-axis in color. EUS-BD is favored regardless of the expected re-intervention rate within the evaluated range of 0%-50% for either procedure, due to the extent of cost-savings with EUS-BD compared to PTBD. EUS-BD, endoscopic ultrasound-guided biliary drainage; PTBD, percutaneous transhepatic biliary drainage; ERCP, endoscopic retrograde cholangiopancreatography.

From a US Medicare perspective, choosing EUS-BD costs $9,497.03 (95% confidence interval $8,697.70-$10,492.23) and PTBD costs $13,878.44 (95% confidence interval $13,712.44-$14,040.07) to the insurer. Thus, EUS-BD is associated with an average cost-savings of $4,381.41 to the insurer compared to choosing PTBD (**Figure 1**). EUS-BD is favored regardless of the expected re-intervention rate within the evaluated range of 0%-50% for either procedure, due to the extent of cost-savings with EUS-BD compared to PTBD in sensitivity analysis (**Figure 2**).

## Discussion

We performed a cost analysis of EUS-BD and PTBD after failed ERCP in MDBO patients in two national healthcare systems with distinct hospital and physician reimbursement paradigms. In both Korea and the US, the total economic costs were greater in choosing PTBD compared to EUS-BD.

EUS-BD has become a technically feasible and effective BD method. One of the main advantages of EUS-BD over PTBD is that it can be performed in the same session after failed ERCP, thus avoiding delay in treatment (9). For PTBD, although initial PTBD may be done on the same day after failed ERCP, subsequent stent insertion is usually done at another session in different day, and the removal of the catheter may need to be done at yet another session (14). Although a one-step percutaneous stent insertion has been introduced which may be done the same day after failed ERCP, the external drainage catheter is removed at another session in a different day, after resolution of cholestasis and confirmation of stent patency (15). Therefore, 2 to 3 steps of intervention are needed in PTBD and subsequent stent insertion, thus increasing the total cost, especially in outpatient-basis US health care system because a procedure performed in a different day could add to the cost compared to that done on the same day, based on how procedures are reimbursed in the US; addition of extra hospital days would increase the cost as well. In addition, EUS-BD was associated with decreased frequency of unscheduled re-intervention compared to PTBD (8, 9). The reduction of the number of BD procedures and re-intervention in EUS-BD compared to PTBD during the clinical course would result in cost savings of EUS-BD in our study. Indeed, in a recent study using the Medicare database, 24.5% of interventional radiology after failed index ERCP incurred downstream procedure costs compared to 8.7% of ERCP repeat procedures, resulting in a higher mean downstream costs of $8,258 ± 10,596 vs. $5,234 ± 6,275 (P=.0255) (6). In a decision-analysis model for EUS-BD in the US using Medicare’s 2012 professional and facility fees for metropolitan Boston (16), the cost of managing malignant biliary obstruction after failed ERCP was $3,249 for direct access extrahepatic EUS-guided cholangiography and $4,111 for PTBD-based strategy. Another cost-effectiveness analysis using a decision analytic Markov model showed that although the charges associated with EUS-BD index procedure was higher than those of PTBD ($7,391 ± 3,791 per patient vs. $3,578 ± 1,699 per patient), the charges associated with EUS-BD reintervention was less than those of PTBD ($1,648 per patient vs. $50,612 per patient) (P<.001) (17). One retrospective study compared EUS-BD and PTBD after failed ERCP and showed that PTBD was associated with higher adverse event rate and cost (12). However, patients who failed EUS-BD were sent for PTBD, and the most commonly used stents were 10-F plastic stents, which are likely to have shorter patency compared to metal stents. Another retrospective study compared EUS-BD and PTBD with similar results (18). However, it is unclear whether those who underwent PTBD received subsequent biliary stent insertion. Since both studies are retrospective, certain limitations exist in cost analysis. However, our study is the first to provide generalizable data in two countries based on national costs data appropriate to the different medical insurance programs and patient management in Korea (fee-for-service and length of hospital stay) and the US (capitated payment and timing/frequency of consultation).

In our cost analysis, we found that EUS-BD generated lower costs to national insurers in both countries compared to PTBD, recognizing that the insurance perspective translates to hospital reimbursement. From a hospital perspective, offering EUS-BD as standard should also consider (1) the marginal economic and clinical impact of deferring other outpatient procedures in order to offer EUS-BD routinely and (2) the costs to purchase clinically appropriate stents necessary to perform EUS-BD. Recognizing that the hospital perspective depends highly on these local factors, it is possible that stent prices from manufacturers and competing patient needs represent barriers to broader and routine adoption of EUS-BD (19). Furthermore, less availability of expert endosonographers compared to interventional radiologists may be the hurdle for widespread utilization of EUS-BD rather than the cost in some centers. However, taken together cost-saving of EUS-BD in the present study, and high patient preference (circa 80%) of EUS-BD over PTBD after failed ERCP in previous international multicenter survey (10), EUS-BD may be more widely used when EUS-BD in outpatient basis is available.

Lastly, as the value of patient care - which is often defined as clinical outcome over cost - may dictate the healthcare reimbursement rate, the clinical implication has a direct impact on the financial health of a healthcare institution. Largest studies (n≥60) comparing outcomes of EUS-BD and PTBD is summarized in **Table 4**. Based on these results (8, 12, 18, 20), EUS-BD provides sanguine clinical advantages over PTBD in the management of MDBO. As patient safety is one of two main components of clinical outcome (efficacy being the other), a treatment modality that offers lower frequencies of adverse outcomes – including procedural complications and unexpected need for re-intervention – ought to be considered superior compared to other methods. Given that EUS-BD offers superior adverse outcome profiles, its advocacy extends beyond a financial benefit of being “less expensive,” to delivering a superior value proposition.

**Table 4:** Summary of largest comparative studies (n ≥ 60) on outcomes of EUS-guided biliary drainage and percutaneous transhepatic biliary drainage.

	Study design	Patient number (n)	Technical success in EUS-BD vs. PTBD, % (n)	Clinical success in EUS-BD vs. PTBD, % (n)	Adverse events in EUS-BD vs. PTBD, % (n)	Reintervention rate in EUS-BD vs. PTBD, % (n)
Khashab et al. (12)	Retrospective	73	86.4 (19/22) vs. 100 (51/51)	100 (19/19) vs. 86.4 (47/51)	18.2 (4/22) vs. 39.2 (36/51)	15.8 (3/19) vs. 43.1 (23/51)
Sharaiha et al. (20)	Retrospective	60	91.6 (43/47) vs. 93.3 (12/13)	62.2 (29/47) vs. 25 (3/13)	6.6 (3/47) vs. 53.8 (7/13)	1.3 vs. 4.9 (mean frequency)
Lee et al. (8)	Randomized trial	66	94.1 (32/34) vs. 96.9 (31/32)	87.5 (28/32) vs. 87.1 (27/31)	8.8 (3/34) vs. 31.2 (10/32)	25 (8/32) vs. 54.8 (17/31)
Téllez-Ávila et al. (18)	Retrospective	62	90 (27/30) vs. 78 (25/32)	96 (29/30) vs. 63 (20/32)	6.6 (2/30) vs. 28 (9/32)	NA

EUS-BD, EUS-guided biliary drainage; PTBD, percutaneous transhepatic biliary drainage; NA, not available.

The strength of the study is that cost comparison was done in two countries with different health care systems and medical costs. In addition, the costs of the Korean study were collected from a randomized controlled trial (8), which is more robust than the data from retrospective studies (6, 16, 17). We believe this is the first cost-minimization analysis from a multicenter prospective randomized study. Since the technical/clinical success is similar between EUS-BD and PTBD, we performed cost-minimization study rather than cost-effectiveness study.

The limitations of our study are as follows. As the initial Korean study was designed to compare the technical success rates of EUS-BD and PTBD, the sample size might not have been adequate for cost comparison (8). In order to mitigate this limitation, we performed bootstrap sampling analysis, which showed consistent results. In the analysis of the Korean data, indirect medical costs such as cost of hiring a caregiver or other social expenses were not considered. However, as both BD strategies would require similar post-procedure care, indirect costs are likely to be higher with PTBD with frequent scheduled or unscheduled re-intervention and prolonged hospital stay. A systematic review and meta-analysis of the comparison of EUS-BD with PTBD after ERCP failure reported that EUS-BD was associated with lower rate of intervention and more cost-effective (9), which is in agreement with our results. In the US cost study, because this is a reimbursement analysis, the indication does not affect reimbursement (reimbursement is based on the procedure performed and not the indication) and therefore is not included directly in the model. Theoretically, the indication for the procedure and patient anatomy might alter expected rates of re-intervention following an attempted EUS-BD or PTBD. However, this did not have a significant impact on cost-savings found with EUS-BD compared to PTBD in the US cost study. Furthermore, we recognized that performing a cost study at all would require a standard management algorithm with contingency plans for each scenario; these algorithms were developed based on consensus among a non-inclusive international group of advanced endoscopists (study authors), recognizing that individual circumstances may vary outside the scope of this study (and outside the scope of choosing between EUS-BD and PTBD). We also assumed similar clinical outcomes in the US based on the same clinical trial data performed in Korea, recognizing the relative paucity of US data on this entity and likely similarity in technical performance of EUS-BD and PTBD between both countries.

In conclusion, we found that EUS-BD resulted in an impact on cost-savings compared to PTBD by reducing the number of re-intervention and was favored regardless of the expected re-intervention rate in the management of MDBO after failed ERCP. Therefore, where the expertise for EUS-BD is available, the use of EUS-BD rather than PTBD after failed ERCP may represent an efficient use of the health care system in patient management (length of hospital stay or timing/frequency of consultation), even in different medical insurance programs (fee-for-service or capitated payment).

## Data Availability Statement

The raw data supporting the conclusions of this article will be made available by the authors, without undue reservation.

## Ethics Statement

Ethical review and approval was not required for the study on human participants in accordance with the local legislation and institutional requirements. Written informed consent for participation was not required for this study in accordance with the national legislation and the institutional requirements.

## Author Contributions

Study conception and design, DHP, WJY, THL, EDS, and RL. Recruitment of participants and aquisition of data, WJY, THL, and EDS. Financial support, DHP. Analysis and interpretation of data, WJY, THL, and EDS. Drafting of the manuscript, WJY, DHP, EDS, and RL. Critical revision of manuscript for important intellectual content, SJ, DHP, and RL. Study supervision, DHP. All authors contributed to the article and approved the submitted version.

## Funding

This work was supported by the Korea Medical Device Development Fund grant funded by the Korean government (the Ministry of Science and ICT, the Ministry of Trade, Industry and Energy, the Ministry of Health and Welfare, Republic of Korea, the Ministry of Food and Drug Safety) (Project Number: KMDF_PR_20200901_0266, 1711138598), and by Basic Science Research Program through the National Research Foundation of Korea funded by the Ministry of Education (2021R1A6A1A03040260).

## Conflict of Interest

EDS is a consultant for Laborie GI Supply and received travel reimbursement from Bausch Health outside the current work. RL is a consultant for Olympus, ConMed and Medtronic. DHP is an inventor of an issued patent related to DEUS stent that is owned by Asan Foundation and Standard Sci-Tech Inc.

The remaining authors declare that the research was conducted in the absence of any commercial or financial relationships that could be construed as a potential conflict of interest.

## Publisher’s Note

All claims expressed in this article are solely those of the authors and do not necessarily represent those of their affiliated organizations, or those of the publisher, the editors and the reviewers. Any product that may be evaluated in this article, or claim that may be made by its manufacturer, is not guaranteed or endorsed by the publisher.

## Abbreviations

EUS, endoscopic ultrasound; EUS-BD, EUS-guided biliary drainage; PTBD, percutaneous transhepatic biliary drainage; ERCP, endoscopic retrograde cholangiopancreatography.
